# Rutin mediated targeting of signaling machinery in cancer cells

**DOI:** 10.1186/s12935-014-0124-6

**Published:** 2014-11-30

**Authors:** Aliye Aras Perk, Iryna Shatynska-Mytsyk, Yusuf Can Gerçek, Kadir Boztaş, Mevzule Yazgan, Sundas Fayyaz, Ammad Ahmad Farooqi

**Affiliations:** Faculty of Science, Department of Biology, Division of Botany, Istanbul University, Istanbul, 34460 Turkey; Diagnostic Imaging and Radiation Therapy Department, Lviv National Medical University, Lviv, Ukraine; Laboratory for Translational Oncology and Personalized Medicine, Rashid Latif Medical College, 35 Km Ferozepur Road, Lahore, Pakistan

**Keywords:** Signaling, Cancer, Apoptosis

## Abstract

Progress in our understanding of molecular oncology has started to shed light on dysregulation of spatio-temporally controlled signaling pathways, inactivation of tumor suppressor genes, tumour and normal stem cell quiescence, overexpression of oncogenes, extracellular and stromal microenvironments, epigenetics and autophagy. Sequentially and characteristically it has been shown that cancer cells acquire the ability to escape from apoptotic cell death, proliferate uncontrollably, sustain angiogenesis and tactfully reconstitute intracellular pathways to avoid immune surveillance. We have attempted to provide a recent snapshot of most recent progress with emphasis on how rutin modulates wide ranging intracellular signaling cascades as evidenced by in-vitro and in-vivo research. It is worth describing that 'single-cell proteomics' analysis has further improved our understanding regarding intracellular signaling pathways frequently activated in cancer cells resistant to therapeutics and can provide biomarkers for cancer diagnosis and prognosis. Data obtained from preclinical studies will prove to be helpful for scientists to bridge basic and translational studies.

## Introduction

Decades of research have using high-throughput technologies have sequentially revealed that cancer is a multifaceted and genomically complex disease. Genetic, genomic and proteomic studies have provided near complete resolution of landscape of molecular oncology. It is now progressively becoming more clear that inactivation of tumor suppressor genes, overexpression of oncogenes, genomic instability, genetic/epigenetic mutations, tumor microenvironment, intracellular signaling cascades and loss of apoptosis are some of the extensively studied mechanisms. Death receptor pathway is a complicated biological mechanism that initiates by the binding of extracellular ligands such as FasL and TRAIL to respective transmembrane receptors [1]. Ligands signal through the receptors and trigger intracellularly assembly of death domain adaptor protein, FADD and procaspase-8 at receptor to form death inducing signaling complexes (DISC). Activated caspase-8 further activates caspase-3, which is an essential step of extrinsic pathway. Intrinsic pathway operates through transmigration of Bid after caspase-8 mediated cleavage into mitochondrion, thus facilitating release of cytochrome-c, SMAC/DIABLA, Omi/Htra. Mechanistically it has been shown that a signalosome is formed by assembly of Cytochrome C, APAF and Pro-caspase-9 to form a apoptosome. Activated caspase-9 further activated caspase-3 [2,3].

There is a list of newly emerging scientific evidence highlighting molecular mechanisms reported to be modulated by rutin to induce apoptosis in cancer cells. We partition this commentary into in-vitro and in-vivo evidences which have added new layers of knowledge into the existing pool of scientific information related to rutin.

### In vitro studies

Rutin present in curry leaf *Murraya koenigii* extracts is an active ingredient and has significant activity against breast cancer MDA-MB-231 cells [4]. DNA protective effects of rutin against pro-carcinogens in HTC hepatic cells are also reported [5]. We divide this multi-component discussion into how rutin modulates, Wnt signaling, JAK-STAT signaling, EGF signaling, AP-1, NF-κB and Akt. We also discuss how ER stress induced response is targeted by rutin to induce apoptosis in cancer cells.

### Wnt signaling

Binding of WNT protein to the receptor complex initiates a signaling cascade intracellularly. Negative modulators of beta-catenin including CK1 (casein kinase 1), GSK3beta, AXIN1 and APC are inhibited and β-catenin moves into the nucleus to form an active transcription factor complex with TCF to transcriptionally upregulate target genes [6,7].

*Hammada scoparia* flavonoidic fraction and its bioactive ingredient rutin exerted inhibitory effects on survival of leukemic progenitors (CD34(+)38(-)123(+)). Moreover, there was a notable decrease in active glycogen synthease kinase 3 β (GSK-3β) in rutin treated cells [8]. Wolfberry water soluble phytochemicals specifically rutin and quercetin have been shown to stabilize β-catenin in Jurkat cells. Detailed in-vitro analysis indicated an increase in β-catenin protein alongwith a dose-dependent decrease in phosphorylation of GSK-3β on Ser9 in Jurkat cells [9] Shown in figure. Although Rutin did not target Wnt/β-catenin signaling in an experimental model of Xenopus embryos [10], it still needs research in different cancer cell lines.

Figure showing Wnt induced signaling. Rutin has been shown to target different modulators of Wnt signaling.

### JAK-STAT signaling

It is now well established that cytokine-receptor-associated Janus-family kinases (JAKs) phosphorylate intracellularly located, cytoplasmic tails of the receptor to provide docking sites for positioning of monomeric STATs. These receptor docked STATs are phosphorylated and transported into the nucleus to trigger expression of target genes. Increasingly it is being realized that STAT3 mediated signaling is negatively modulated by different inhibitory molecules. Rutin has been shown to inhibit inflammatory responses in UVB-irradiated mouse skin by inhibiting the increase in phosphorylated levels of STAT3 [11].

Therapeutically controlling STAT3 signaling using different natural phytochemicals although has shown promise [12-14] however it still needs a detailed research.

### EGFR induced signaling

Epidermal Growth Factor (EGF) induced signaling has emerged as a deeply studied molecular mechanism. It is intriguing to note that EGF signaling is frequently deregulated in different cancers [15-17]. EGF signals through EGFR in cancer cells. EGFR undergoes auto-phosphorylation at tyrosine residues and is reported to be involved in activating various downstream effectors of different signaling axis particularly, Ras/Raf/Mek/Erk and PI3K/Akt. Rutin has been shown to inhibit EGFR kinase activity. Rutin also exerted inhibitory effects on Akt and Raf/MEK/ERK signaling pathways. Rutin has been noted to directly bind with EGFR as evidenced by pull-down assay which indicated that EGFR protein was pulled down with rutin–Sepharose 4B beads [18]. This finding needs additional verification in different cancer cell lines to know if Rutin can effectively inhibit EGFR induced signaling in HER2-overexpressing breast cancer. Moreover, how effectively rutin may inhibit PDGFR and VEGFR is also an outstanding question that needs to be addressed.

### AP-1, NF-κB and Akt

Activator proteins (AP-1) include the JUN, FOS, ATF protein families, which undergo homo-dimerization and hetero- dimerization through their leucine-zipper domains [19]. AP-1activity has been reported to be modulated by extracellular signals including growth factors and intracellular signaling primarily through extracellular-signal-regulated kinase (ERK), p38 and c-Jun N terminal Kinase [20].

Inhibitor of -Kappa B (IκB) bound Nuclear factor-Kappa B (NF-κB) exists in an inactive state in the cytoplasm, however, proteasomal degradation of IκB promotes its nuclear accumulation to trigger expression of antiapoptotic genes [21].

Transactivation of AP-1 and NF-κB was also notably reduced in rutin treated cells [18]. Wolfberry water soluble phytochemicals specifically rutin and quercetin have considerable biological activity against jurkat cells. Results revealed inhibition of NFκB and AKT activity in jurkat cells [9]. Rutin also inhibited inflammatory responses in UVB-irradiated mouse skin by inhibiting the increase in phosphorylated levels of p38 MAPK and JNK. Moreover, AP-1 did not show nuclear accumulation in rutin treated cells [11].

### Endoplasmic reticulum (ER) stress

The endoplasmic reticulum (ER) stress triggers activation of multifunctional sensors including activating transcription factor 6 (ATF6), inositol-requiring protein 1 (IRE1) and PKR-like ER kinase (PERK) to transduce information about the folding status [22]. Signaling machinery consisting of IRE1 and PERK undergo oligomerization in the plane of the membrane and activated by trans-autophosphorylation of activation loop during ER stress [23]. Some other well studied triggers for ER stress response include loss of binding to BIP (chaperone immunoglobulin heavy chain-binding protein) and intricate interaction with misfolded proteins [23]. Mechanistically, rutin was reported to considerably reduce ROS, IRE1, PERK and ATF6 to induce apoptosis in cancer cells. Gene silencing strategy also confirmed that PERK, ATF6 and IRE1 silenced cancer cells displayed a higher apoptotic rate [9].

### Extrinsic and Intrinsic pathway

BCL2/BAX ratio and expression of BCL2, both were notably reduced in rutin treated neuroblastoma LAN-5 cells. G2/M arrest and a marked increase in apoptotic rate were noted in neuroblastoma LAN-5 cells [24]. Rutin present in ethanolic extract of aerial parts of *Pupalia lappacea* also exerted effects on leukemia K562 cells by functionalizing intrinsic pathway mediated apoptosis [25]. Extract of *Cyrtosperma johnstonii* contains rutin as a bioactive ingredient and has potent biological activity against small cell lung carcinoma cells as evidenced by cell cycle arrest and apoptosis [26].

### In vivo

Rutin considerably reduced tumor growth in mice xenografted with SW480 colon cancer cells [27]. Extract of *Phyllanthus urinaria* is rich in polyphenols particularly rutin. Extract has been shown to remarkably inhibit tumor spread in mice xenografted with metastatic A549 and Lewis lung carcinoma (LLC) cells. Mechanistically it was shown that nuclear accumulation of NF-κB and AP-1 was drastically reduced. Moreover, metalloproteinase-2 expression was also noted to be downregulated [28]. It is noteworthy that administration of 120 mg/kg of rutin in mice xenografted with leukemia HL-60 cells induced regression of tumor [29]. Radioprotective effects of troxerutin are also studied in irradiated mice [30]. Extract of *Prunella vulgaris* is enriched in rosmarinic acid, quercetin and rutin. Extract significantly inhibited tumor growth in C57BL/6 mice [31]. It has previously been convincingly revealed that rutin remarkably reduced size of enlarged spleen in mice intraperitoneally injected with WEHI-3 cells [32]. Rutin has been shown experimentally to effectively block development of adenomas in the lungs of wild-type mice, however the results were not noted in BRM null mice [33]. It has recently been reported that preneoplasic lesions induced by 1,2-dimethylhydrazine in rat colon were remarkably reduced in troxerutin treated animal group [34].

There is a direct piece of experimental evidence suggesting that rutin mediated suppression in monocyte migration into peritoneal tumors contributes to tumor growth. The results revealed that macrophages infiltrating tumor dramatically reduced peritoneal colorectal carcinoma metastases however rutin significantly inhibited infiltration of macrophages [35].

### Human studies

Pharmacokinetic profile of rutin was determined in 18 healthy non-obese females having normal cholesterol levels who volunteered for the study. Plasma flavonoids were considerably higher in the rutin-supplemented females. Endogenous oxidation of pyrimidines was significantly decreased in both placebo and rutin-treated volunteers [36]. In another, diet-controlled, double blind two-period cross-over study, 16 healthy volunteers were orally administered with varying doses of rutin. After rutin ingestion, inter-individual variability in maximum concentration (Cmax) and area under curve (AUC) (0-32) values were significant and gender associated [37]. Mesenteric lymphatic/duodenum-cannulated rat model was intraduodenally administered with 300 mg/kg of Rutin. Maximum concentration of rutin in lymph, was slightly lesser as compared to plasma. Area under curve (AUC) of rutin in lymph was 2-fold higher as compared to plasma rutin [38].

### Pharmacokinetics

There is a recent report suggesting that phenolic compounds including resveratrol, quercetin, and rutin displayed poor absorption through colon adenocarcinoma Caco-2 cells [39]. Different approaches have been utilized to enhance availability of rutin and in line with this approach, encapsulation of rutin in different substituents of cyclodextrin, such as 2-hydroxypropyl-β-cyclodextrin (HP-β-CD), hydroxypropyl-γ-cyclodextrin(HP-γ-CD),β-cyclodextrin (β-CD) and γ2-β-cyclodextrin(γ2-β-CyD) have shown potential in improving solubility and stability of rutin [40]. Mechanistically it has been shown that β-CD and HP-β-CD formed stable inclusion complexes with rutin [41]. Rutin dissolution rates enhanced efficiently upon complexation with cyclodextrins. Cyclodextrins stabilize rutin in gastrointestinal tract (GIT) after oral administration as rutin hydrolysis in small intestinal homogenates of drug treated animal group was considerably reduced. Oral bioavailability of rutin has also been noted to be significantly increased upon complexation with HP-β-CyD as evidenced by faster dissolution rate, increase in solubility and gastrointestinal stability. Higher aqueous solubility and negligible toxicity is a hallmark of HP-β-CyD associated pharmaceutical formulations.

Absorption of rutin from the gastrointestinal tract (GIT) is slower. Cross-linked sodium carboxy, methylcellulose (CMC-XL) has been used to formulate rutin containing fast-release tablets and prolonged-release formulations using hydroxypropylmethylcellulose (HPMC) of different viscosity grades have also been developed [42]. There is an exciting piece of evidence highlighting that hydrolyzed rutin had a higher biological activity against wide ranging cancer cell lines [43].

### Rutin regulation of DNA damage

Ethyl methanesulfonate (EMS) induced alkylation mediated DNA damage was notably reduced in Drosophila melanogaster males because rosmarinic acid and rutin encircled nucleotides and occupied EMS binding space thus generating an impermeable barrier for the EMS molecule to trigger alkylation [44]. Moreover, Doxorubicin induced DNA damage was notably reduced in Rutin treated hepatoma HepG2 cells [45]. However, another role of Rutin has been documented as a DNA damage inducer. Rutin moderately induced DNA damage in BRCA mutant cells [46]. The data related to how Rutin actually modulates DNA damage signaling is insufficient and needs detailed research. It will be essential to note how it positively and/or negatively modulates DNA damage signaling in different cancer cell lines.

### Concluding remarks

Although there are advancements in understanding of the molecular networks and signaling cascades reported to be modulated by rutin in cancer cells, it still needs detailed research. TGF/SMAD and SHH mediated signaling axis are insufficiently studied in different cancer cell lines. Moreover, we still have outstanding questions regarding rutin mediated effects on oncogenic and tumor suppressor micro RNAs. Detailed and extensive research should be focused on combinatorial approaches to overcome resistance against therapeutics in resistant phenotypes.
